# Pulsed electromagnetic fields stimulation prevents steroid-induced osteonecrosis in rats

**DOI:** 10.1186/1471-2474-12-215

**Published:** 2011-09-29

**Authors:** Shuai Ding, Hao Peng, Hong-Song Fang, Jian-Lin Zhou, Zhe Wang

**Affiliations:** 1 Department of Orthopedics, Renmin Hospital of Wuhan University, Wuhan 430060, Hubei Province, People's Republic of China

## Abstract

**Background:**

Pulsed electromagnetic fields (PEMF) stimulation has been used successfully to treat nonunion fractures and femoral head osteonecrosis, but relatively little is known about its effects on preventing steroid-induced osteonecrosis. The purpose of the study was to investigate the effects of PEMF stimulation on the prevention of steroid-induced osteonecrosis in rats and explore the underlying mechanisms.

**Methods:**

Seventy-two male adult Wistar rats were divided into three groups and treated as follows. (1) PEMF stimulation group (PEMF group, n = 24): intravenously injected with lipopolysaccharide (LPS, 10 μg/kg) on day 0 and intramuscularly injected with methylprednisolone acetate (MPSL, 20 mg/kg) on days 1, 2 and 3, then subjected to PEMF stimulation 4 h per day for 1 to 8 weeks. (2) Methylprednisolone-treated group (MPSL group, n = 24): injected the same dose of LPS and MPSL as the PEMF group but without exposure to PEMF. (3) Control group (PS group, n = 24): injected 0.9% saline in the same mode at the same time points. The incidence of osteonecrosis, serum lipid levels and the mRNA and protein expression of transforming growth factor β1 (TGF-β1) in the proximal femur were measured 1, 2, 4 and 8 weeks after the last MPSL (or saline) injection.

**Results:**

The incidence of osteonecrosis in the PEMF group (29%) was significantly lower than that observed in the MPSL group (75%), while no osteonecrosis was observed in the PS group. The serum lipid levels were significantly lower in the PEMF and PS groups than in the MPSL group. Compared with the MPSL and PS groups, the mRNA expression of TGF-β1 increased, reaching a peak 1 week after PEMF treatment, and remained high for 4 weeks, then declined at 8 weeks, whereas the protein expression of TGF-β1 increased, reaching a peak at 2 weeks after PEMF treatment, and remained high for 8 weeks.

**Conclusions:**

PEMF stimulation can prevent steroid-induced osteonecrosis in rats, and the underlying mechanisms involve decreased serum lipid levels and increased expression of TGF-β1.

## Background

Osteonecrosis of the femoral head is the end point of a disease process that results in progressive collapse of the femoral head followed by destruction of the hip joint. It has been recognized as a side effect of the corticosteroid used to treat diseases such as Acute Respiratory Syndrome (SARS), Acquired Immunodeficiency Syndrome (AIDS) and Systemic Lupus Erythematosus (SLE) [1-3]. High-dose corticosteroid administration is considered to be the most common risk factor for osteonecrosis [4,5]. With the progression of osteonecrosis, both bone and cartilage tissue are deformed, which ultimately leads to collapse of the load-bearing area of the femoral head. Once osteonecrosis collapses the femoral head, most patients require surgical treatment. Several surgical treatments have been established to prevent collapse, such as core decompression [6], osteotomy [7], vascularized or nonvascularized bone grafting [8] and joint arthroplasty [9]. Most of them have certain effects in selected series, but the costs and complications of surgery cannot be ignored. Therefore, preventing osteonecrosis would be an ideal strategy for the treatment of this disease, but there is no established prophylactic measure.

It is well documented that pulsed electromagnetic fields (PEMF) are useful for enhancing bone repair in nonunion fractures and related bone-healing problems [10]. In addition, it was used successfully for the treatment of osteonecrosis of the femoral head, especially at the early stage [11,12], but relatively little is known about its effects on preventing steroid-induced osteonecrosis. Furthermore, the mechanisms of PEMF stimulation for the prevention of steroid-induced osteonecrosis remained unclear, and the optimal protocol for PEMF should be explored [13]. Transforming growth factor β1 (TGF-β1), involved in bone remodeling, is a multifunctional cytokine. It plays an important role in controlling osteoblast proliferation and differentiation in vivo [14]. Some studies demonstrate that mechanical interruption by lipid emboli in the nutrient vessels can lead to vascular occlusion, and hyperlipidemia has been linked to the development of osteonecrosis [15,16]. We proposed that PEMF may be beneficial in preventing steroid-induced osteonecrosis of the femoral head, and the underlying mechanisms involve decreased serum lipid levels and increased the expression of TGF-β1. As a preventive therapy, PEMF could be used in combination with corticosteroid for treatment of many clinical conditions, such as AIDS and SLE, which require high-dose corticosteroid treatment.

In this study, we investigated the preventive effect of PEMF on steroid-induced osteonecrosis by examining the incidence of osteonecrosis of the femoral head as well as the serum lipid levels and the mRNA and protein expression of TGF-β1.

## Methods

### Animals

Seventy-two male adult Wistar rats (obtained from the experimental animal center of Wuhan University), weighing 250-280 g, were used in this study. All rats were housed individually in custom-designed Plexiglas cages (55 × 35 × 26 cm) under standard laboratory conditions (12/12-h light/dark cycle, 24-25°C, humidity 50-55%) and allowed free access to food and water during the study. All experiment protocols adhered to the Guidelines for the Care and Use of Laboratory Animals published by the U.S. National Institutes of Health (NIH Publication, revised 1996) and approved by the ethics committee for Animal Research, Wuhan University, China.

### Grouping and treatment

All rats were divided into three groups by randomized block design according to weight. (1) PEMF stimulation group (PEMF group, n = 24): intravenously injected with 10 μg/kg lipopolysaccharide (LPS, *Escherichia coli *0111:B4, Sigma-Aldrich, St. Louis, MO, USA) on day 0 and injected with 20 mg/kg methylprednisolone acetate (MPSL, Pfizer Pharmaceutical, China) into the right gluteus medius muscle on days 1, 2 and 3 at a time interval of 24 h [17], then subjected to PEMF stimulation 4 h per day for 1 to 8 weeks from day 4 onward. (2) Methylprednisolone-treated group (MPSL group, n = 24): injected with the same dose of LPS and MPSL as the PEMF group, with no exposure to PEMF. (3) Control group (PS group, n = 24): injected with 0.9% saline in the same mode at the same time points. Six rats in each group were sacrificed, and the samples were collected 1, 2, 4 and 8 weeks after the last MPSL (or saline) injection. The rats were anesthetized using pentobarbital sodium (50 mg/kg ip; Westang Biotechnology, Inc., Shanghai, China). Blood samples were collected from the inferior vena cava with the animals in a fasting state. Then, the rats were sacrificed with an overdose of pentobarbital sodium (240 mg/kg ip) and bilateral femurs were harvested.

### PEMF Generators

**Figure 1:**
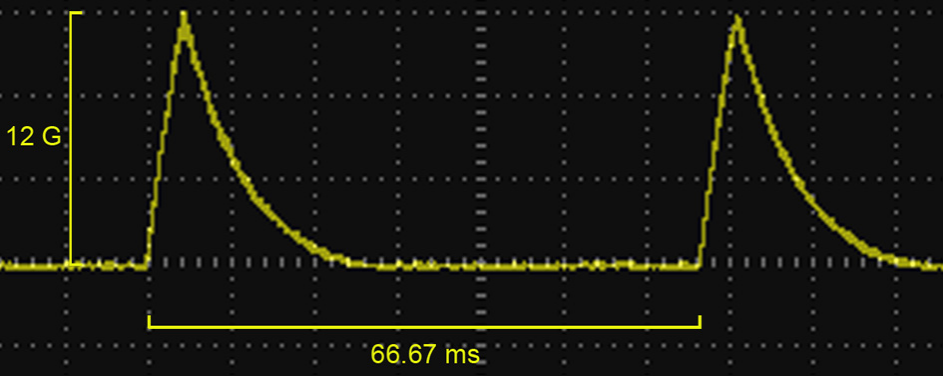
**Waveform of the PEMF**. The frequency of the PEMF was 15 Hz. During each pulse, the magnetic field increased from 0 to 12 G in 4.5 ms and then decreased back to 0 in 20 ms.

The PEMF generators consisted of a signal generator and a pair of 40-cm diameter Helmholtz coils, which were designed and manufactured by the Department of Physics, Wuhan University, China. The Helmholtz coils, each of which contained 500 turns of enameled copper wire with diameter of 0.8 mm, were wound on a nonconducting spool. The coils, equal to the width of the cage, were separately placed at a distance of 25 cm. The coils were connected to a signal generator that delivered repetitive, single, square-wave pulses with pulse duration of 4.5 ms and frequency of 15 Hz. The waveform of the PEMF is presented in Figure 1. The frequency of the PEMF was 15 Hz [13]. During each pulse, the magnetic field increased from 0 to 12 G in 4.5 ms and then decreased back to 0 in 20 ms. The rats in the PEMF group were exposed to active pulsed electromagnetic fields 4 h each day. The other rats were housed in identical cages, but with no stimulation.

### Histopathology

The proximal one-third of right femurs was fixed in 4% paraformaldehyde- 0.1 M phosphate buffer (pH 7.4), followed by decalcification with 10% ethylenediaminetetraacetic acid (EDTA)- 0.1 M phosphate buffer (pH 7.4). After decalcification, the tissues were dehydrated in graded ethanol, embedded in paraffin, cut into 5-μm thick sections in the coronal plane, and processed for routine hematoxylin and eosin staining for the evaluation of osteonecrosis. All sections were assessed blindly by two independent authors (SD, HP), and the diagnosis of osteonecrosis was established based on the presence of empty lacunae or pyknotic nuclei of osteocytes in the bone trabeculae, accompanied by surrounding bone marrow cell necrosis [18]. If the diagnoses differed between the two examiners, a consensus was reached by discussing the histologic findings without knowledge of the group from which the sample was obtained. The rats with at least one osteonecrotic lesion in the area examined were considered to have developed osteonecrosis.

### Hematological examination

The serum was collected by centrifugation at 3000 rpm for 10 min at 4°C then stored at -80°C until hematological examination. The levels of triglyceride (TG), total cholesterol (TC), low-density lipoprotein cholesterol (LDL) and high-density lipoprotein cholesterol (HDL) in serum samples were determined by automatic biochemical analyzer (AU 1000, Olympus, Japan).

### Polymerase chain reaction (PCR) analysis

The unfixed proximal one-third of left femurs was quick-frozen in liquid nitrogen and stored at -80°C for subsequent mRNA and protein extraction. Samples were weighed, followed by pulverization with a mortar and pestle under liquid nitrogen in an Rnase-free condition. Total RNA was extracted using Trizol Reagent (Invitrogen, Carlsbad, CA, USA) according to the manufacturer's instructions. The concentration of RNA was quantified by measuring the absorbance at 260 nm (A_260_). The purity of RNA was assessed by determining the A_260_/A_280 _ratio. The integrity and size distribution of RNA were confirmed by formaldehyde-agarose gel electrophoresis and ethidium bromide staining. The RevertAid First Strand cDNA Synthesis Kit (Fermentas Life Sciences, EU) was used to synthesize complementary DNA (cDNA). TGF-β1 cDNA was amplified by PCR using the primers 5'- GGCGGTGCTCGCTTTGTA-3' (forward) and 5'-GCGGGTGACTTCTTTGGC-3' (reverse) (amplification product size 106 bp) [GenBank: NM021578]. β-actin cDNA was amplified as an internal control using the primers 5'-TGGTGGGTATGGGTCAGAAGG-3' (forward) and 5'-ATGGCTGGGGTGTTGAAGGTC-3' (reverse) (amplification product size 265 bp) [GenBank: NM031144]. The cycle parameters of the reaction system included an initial denaturation step of 94°C for 5 min; amplification consisted of 94°C for 30 s, 60°C for 30 s and 72°C for 45 s, followed by a final extension step of 72°C for 10 min. Thirty cycles (β-actin) and thirty-five cycles (TGF-β1) of amplification were performed. PCR amplification products were analyzed by 1.5% agarose gel electrophoresis, stained with ethidium bromide and photographed by the Geliance 200 Imaging System (PerkinElmer, USA). The optical density of the bands was analyzed with Quantity One software (Bio-Rad Laboratories, Hercules, CA). Gene expression was reported as the optical density ratios of TGF-β1 to β-actin.

### Western blot analysis

Samples dissected from the proximal one-third of left femurs were powdered in liquid nitrogen by hand milling, followed by homogenization in ice-cold radioimmunoprecipitation (RIPA, Beyotime institute of Biotechnology, China) buffer containing phenylmethylsulfonyl fluoride (PMSF, Beyotime institute of Biotechnology, China) and a cocktail of protease inhibitors (Complete, EDTA-free; Roche, Mannheim, Germany). After sonication, the samples were centrifuged twice at 14000 rpm at 4°C for 10 min to remove cell debris, nuclei and large particulates. The supernatant containing the cytosolic protein fraction was then collected. A quarter volume of 5 × loading buffer was added and boiled at 95°C for 5 min then stored at -20°C until electrophoresis. Proteins were separated by 10% sodium dodecyl sulfate polyacrylamide gel and transferred to polyvinylidene difluoride membranes (Millipore Corporation, Bedford, MA). After being blocked with 2% bovine serum albumin (Roche, Mannheim, Germany), the membrane was incubated at 4°C overnight with rabbit anti-TGF-β1 antibody (1:500, Santa Cruz Biotechnology, Santa Cruz, CA) or rabbit anti-β-actin antibody (1:500, Santa Cruz Biotechnology, Santa Cruz, CA) as primary antibodies, followed by exposure to peroxidase-conjugated secondary antibodies (1:2000, Jackson Laboratories, West Grove, PA, USA) at 25°C for 1 h. The proteins on the membrane were visualized using an ECL plus detection kit (Amersham Pharmacia Biotech, Buchinghamshire, UK), exposed to Kodak X-ray film, then photographed by the Geliance 200 Imaging System. The optical density of the bands was analyzed with Quantity One software. The expression of TGF-β1 was then normalized against β-actin.

### Statistics

All data are presented as the mean ± SD. Statistical analysis was performed using SPSS 13.0 software (SPSS Inc., Chicago, IL, USA). One-way analysis of variance (ANOVA) with Turkey's post hoc test was used to examine differences between groups. Statistical significance was set at *P <*0.05.

## Results

### Incidence of osteonecrosis

**Figure 2:**
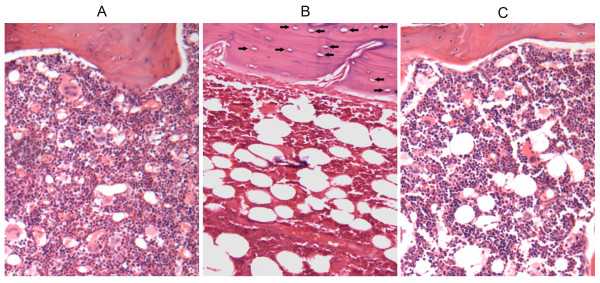
**Histological appearance of the proximal one-third of femur in the three groups at week 8**. (A) PS group. (B) MPSL group. (C) PEMF group. The arrows represent empty lacunae or pyknosis of osteocytes in the bone trabeculae, accompanied by surrounding bone marrow cell necrosis. (hematoxylin and eosin stain, ×100).

All rats survived during the experimental period. Figure 2 shows the histopathological appearance of the proximal one-third of the femur in the three groups after hematoxylin and eosin staining at week 8. The incidence of osteonecrosis is shown in Table 1. The incidence of osteonecrosis was 7/24 in the PEMF group (29%) and 18/24 (75%) in the MPSL group, while no osteonecrosis was observed in the PS group. The incidence of osteonecrosis in the PEMF group was significantly lower than that in the MPSL group (*P <*0.05).

**Table 1:** Incidence of osteonecrosis of the femur (%).

*Groups*	*1 week*	*2 weeks*	*4 weeks*	*8 weeks*	*Total*
PEMF	33	17	33	33	29*
MPSL	50	67	100	83	75
PS	0	0	0	0	0

*P <0.05 versus MPSL group.

### Hematological examination

**Figure 3:**
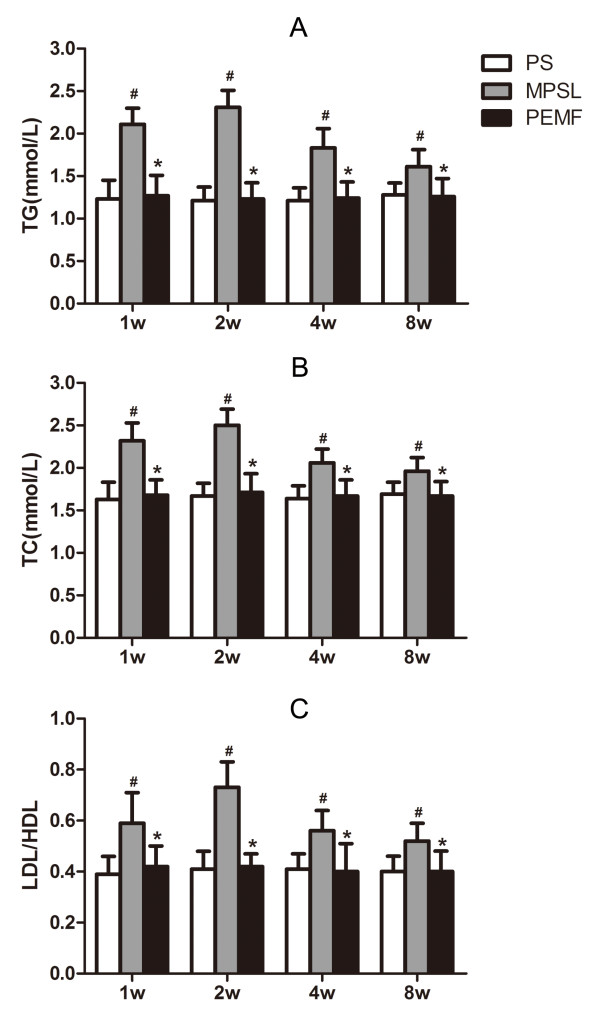
**Hematological examination of rats in the three groups**. (A) TG. (B) TC. (C) LDL/HDL. Data are presented as the mean ± SD. ^#^*P <*0.05 versus PS group, *P <0.05 versus MPSL group.

The levels of TG, TC and LDL/HDL were significantly increased from baseline in the MPSL group (*P <*0.05) from week 1 onward, and the values increased up to a maximum at week 2, then decreased from week 4 onward. No detectable changes were found upon the hematological examinations of the PEMF or PS group. The lipid levels were significantly lower in the PEMF and PS groups than in the MPSL group at all time points (*P <*0.05) (Figure 3A-C).

### mRNA expression of TGF-β1

**Figure 4:**
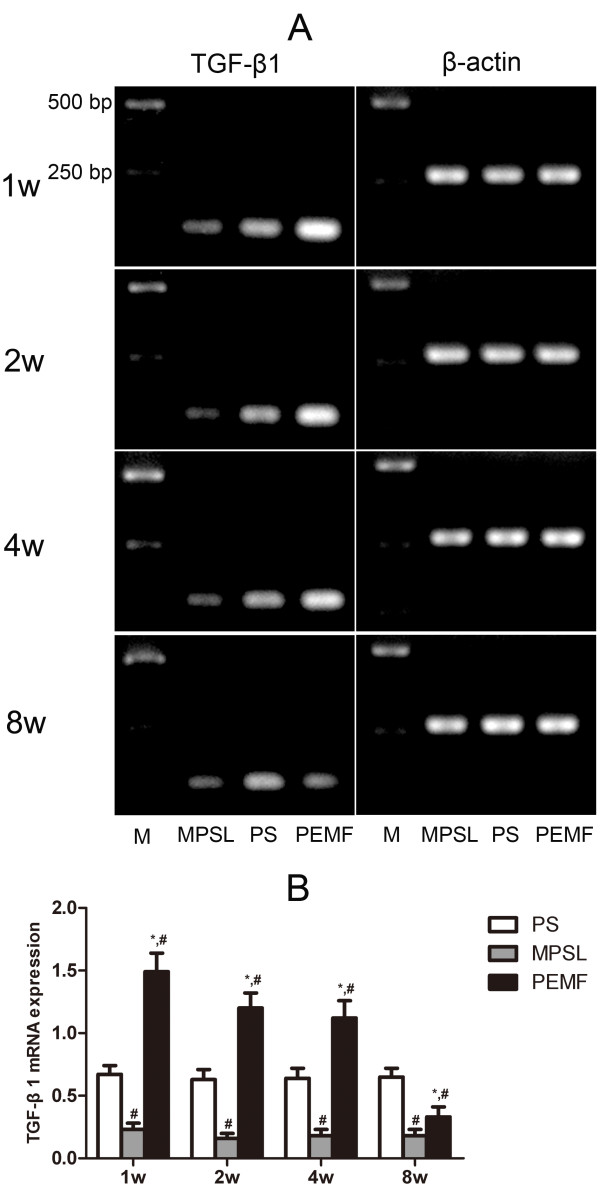
**mRNA expression of TGF-β1 in proximal femur**. (A) mRNA expression levels of TGF-β1 were evaluated by PCR analysis. M: PCR marker, from bottom to top, the size is 250 and 500 bp. (B) Data are expressed as expression ratios normalized to β-actin gene expression and presented as the mean ± SD. ^#^*P <*0.05 versus PS group, *P <0.05 versus MPSL group.

The mRNA expression of TGF-β1 in the PEMF group was significantly higher compared with corresponding samples in the MPSL group at all time points (*P <*0.05), and significantly higher than that in the PS group at 1, 2 and 4 weeks after PEMF stimulation treatment (*P <*0.05). Compared with the PS group, the expression of TGF-β1 in the MPSL group was markedly suppressed (*P <*0.05) (Figure 4A-B).

### Protein expression of TGF-β1

**Figure 5:**
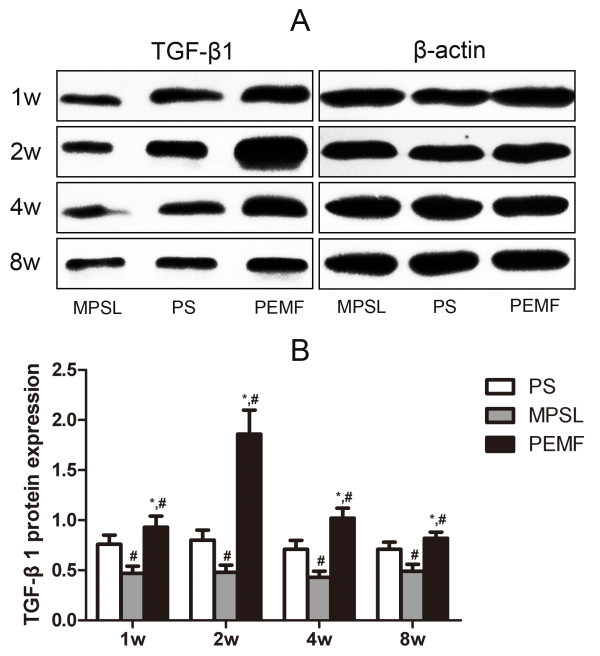
**Protein expression of TGF-β1 in proximal femur**. (A) The protein expression levels of TGF-β1 were evaluated by western blot analysis. (B) Data are expressed as expression ratios normalized to β-actin protein expression and presented as the mean ± SD. ^#^*P <*0.05 versus PS group, *P <0.05 versus MPSL group.

Figure 5 shows the protein expression of TGF-β1 in the proximal femur. The protein expression of TGF-β1 was markedly higher in the PEMF group than in the MPSL and PS groups at all time points (*P <*0.05). In contrast, the protein expression of TGF-β1 was significantly lower in the MPSL group than in the other two groups (*P <*0.05).

## Discussion

PEMF stimulation has been used successfully to treat nonunion fractures and osteonecrosis of the femoral head, but relatively little is known about its effects on preventing steroid-induced osteonecrosis. Furthermore, the mechanisms and the optimal protocol for PEMF stimulation for the prevention of steroid-induced osteonecrosis remain unclear. We investigated the preventive effect of PEMF on steroid-induced osteonecrosis in rats and explored the underlying mechanisms.

In the present study, the steroid-induced osteonecrosis model was established in rats by a single injection of low-dose LPS combined with three subsequent injections of high-dose MPSL [17]. The histopathology of osteonecrosis generated in this model was characterized by empty lacunae or pyknotic nuclei of osteocytes accompanied by surrounding bone marrow cell necrosis, which was similar to osteonecrosis in steroid- treated patients. However, there are some differences in the osteonecrosis observed in rats as compared to humans. For example, osteonecrosis often leads to femoral head collapse in humans but not in rats, as the epiphyseal line of the femur is permanent in adult rats but not in adult humans. In addition, the metabolic rates are higher in rats than in humans, so osteonecrosis can be observed in rats within one week after steroid treatment [19]. In the present study, the incidence of osteonecrosis (in the MPSL group) was 75%, and no rats died during the experimental period, indicating that this rat model is safe and effective. Thus, this model is useful for steroid-induced osteonecrosis studies.

Since Bassett et al. [20] first reported the therapeutic effects of PEMF in bone healing in 1974, the treatment was widely used in nonunion fractures and related bone healing problems. Different parameters of PEMF, such as frequency, intensity and stimulation time, were manipulated in clinical and experimental studies. However, the optimal parameters of PEMF are not known. In this study, the choice of PEMF parameters was based on a report by Ishida et al. [13], in which they found that PEMF could reduce the risk of steroid-induced osteonecrosis. We used the same electromagnetic frequency, changed the stimulation time from 10 h to 4 h per day, and obtained a similar incidence of osteonecrosis.

The incidence of osteonecrosis in the PEMF group was markedly lower than in the corresponding samples in the MPSL group at all time points. The total incidence of osteonecrosis was 29% versus 75% in the two groups, respectively. Several researchers have shown that steroid-induced osteonecrosis can be prevented by anticoagulants or lipid-lowering agents, with reductions in osteonecrosis that range from 30% to 40% [21,22]. Our study showed similar prevention effects, and no rat suffered tissue damage due to the PEMF treatment. Therefore, PEMF stimulation is a safe and effective treatment for preventing osteonecrosis. We also found that the incidence of osteonecrosis in the MPSL group displayed a progression, while that in the PEMF group did not. In the MPSL group, the incidence of osteonecrosis increased to a peak at 4 weeks after the last MPSL injection and then declined at 8 weeks. It is interesting that the serum lipid levels in the MPSL group showed a similar trend, increasing to a peak at 2 weeks after the last MPSL injection and declining at 4 weeks. This finding might indicate that hyperlipidemia contributes to the pathogenesis of osteonecrosis.

Steroid treatment could increase adipogenesis and decrease type-I collagen and ostecocalcin mRNA expression [5]. In addition, lipid-lowering agents were used to prevent osteonecrosis and revealed satisfactory results [21,22]. The data indicated that hyperlipidemia may be one of the pathological mechanisms of steroid-induced osteonecrosis. Therefore, the prevention of hyperlipidemia might also prevent steroid-induced osteonecrosis. We consider that PEMF could prevent osteonecrosis because several studies have shown that it can decrease serum lipid levels [12,23]. Ishida et al. [13] also found that PEMF did not affect bone marrow fat cell size and hypothesized that its preventative effect on steroid-induced osteonecrosis occurs via a mechanism independent of lipid metabolism. Therefore, in the present study, we measured only serum markers of adipogenesis, including TG, TC, LDL and HDL, in order to explain the underlying mechanism of PEMF stimulation in preventing steroid-induced osteonecrosis. Assays of TG, TC and LDL/HDL in the MPSL group showed a significant increase compared with the PEMF and PS groups at all time points. In contrast, the serum lipid levels were similar between the PEMF group and the PS group. Thus, we speculated that PEMF stimulation may prevent osteonecrosis by decreasing serum lipid levels. The underlying mechanism of PEMF in the living organism remains unclear. Some theories have been proposed: that the electromagnetic fields have the potential to regulate flow through cation channels, changing the steady-state concentrations of cellular cations and thus the metabolic processes dependent on cation concentrations. We therefore hypothesize that the biological effects of PEMF on serum lipids were associated with ion-channel gating on the cell membrane [24,25].

As shown in previous studies, TGF-β1 is involved in many aspects of skeletal development and regulation, such as fracture repair and bone regeneration, as it can promote the proliferation and differentiation of osteoblasts [14]. The results of this study showed that the mRNA and protein expression of TGF-β1 was suppressed in the MPSL group but up-regulated in the PEMF group. Our findings are consistent with previous studies, which reported that the expression of TGF-β1 could be enhanced through use of an electromagnetic field (EMF) [26]. Therefore, it is highly likely that the positive effect of PEMF stimulation on the expression of TGF-β1 in the proximal femur of steroid-treated rats contributes to the prevention of steroid-induced osteonecrosis.

Although the pathophysiology of osteonecrosis of the femoral head has not been completely elucidated, high-dose corticosteroid administration is considered as the most common risk factor for osteonecrosis [4,5]. Dosages typically considered to be associated with the disease are > 2 g of prednisone, or its equivalent, within a period of two to three months. Lower dosages are not typically related to osteonecrosis of the femoral head [4]. As a preventive therapy, PEMF could be used in combination with corticosteroid for treatment of many clinical conditions, such as AIDS and SLE, which require high-dose corticosteroid treatment. Daily treatment of PEMF is both time consuming and demanding; patients may find it preferable to perform the treatment at night. During the treatment, the coils were installed separately on the sides of the bed to generate an electromagnetic field on the gluteofemoral area.

One limitation of this study is that we measured only serum markers of adipogenesis, including TG, TC, LDL and HDL, but did not evaluate the direct effect of PEMF stimulation on adipogenesis in steroid-treated rats. The other is that X-ray images were not used for the evaluation of osteonecrosis.

## Conclusions

In summary, PEMF stimulation can prevent steroid-induced osteonecrosis in rats, and the underlying mechanisms involve decreased serum lipid levels and increased expression of TGF-β1. Because PEMF stimulation is effective, safe and noninvasive, it provides a useful prophylaxis for steroid-induced osteonecrosis.

## Competing interests

The authors declare that they have no competing interests.

## Authors' contributions

SD, HP and JLZ were involved in the design of the study. SD, HP and HSF carried out the histopathological and hematological analysis. JLZ and ZW carried out the molecular analysis. SD and HSF performed the statistical analysis. SD and HP drafted the manuscript. All authors read and approved the final manuscript.

## Pre-publication history

The pre-publication history for this paper can be accessed here:

http://www.biomedcentral.com/1471-2474/12/215/prepub
